# “Danmu” preference, problematic online video watching, loneliness and personality: An eye-tracking study and survey study

**DOI:** 10.1186/s12888-023-05018-x

**Published:** 2023-07-20

**Authors:** Zhihao Yan, Zeyang Yang, Mark D. Griffiths

**Affiliations:** 1 Department of Psychology, School of Education, Soochow University, Suzhou, 215123 China; 2 School of Educational Science, Anhui Normal University, Wuhu, China; 3 International Gaming Research Unit, Psychology Department, Nottingham Trent University, Nottingham, UK

**Keywords:** Danmu, Eye-tracking, Problematic online video watching, Loneliness, Personality

## Abstract

‘Danmu’ (i.e., comments that scroll across online videos), has become popular on several Asian online video platforms. Two studies were conducted to investigate the relationships between Danmu preference, problematic online video watching, loneliness and personality. Study 1 collected self-report data on the study variables from 316 participants. Study 2 collected eye-tracking data of Danmu fixation (duration, count, and the percentages) from 87 participants who watched videos. Results show that fixation on Danmu was significantly correlated with problematic online video watching, loneliness, and neuroticism. Self-reported Danmu preference was positively associated with extraversion, openness, problematic online video watching, and loneliness. The studies indicate the potential negative effects of Danmu preference (e.g., problematic watching and loneliness) during online video watching. The study is one of the first empirical investigations of Danmu and problematic online video watching using eye-tracking software. Online video platforms could consider adding more responsible use messaging relating to Danmu in videos. Such messages may help users to develop healthier online video watching habits.

## Introduction

‘Danmu’ is an emerging type of anonymous comment system found in online videos, in which comments float across the screen from right to left while the videos are playing. The term *‘*Danmu (“bullet curtain” or “barrage” in Chinese) was translated from the Japanese word *danmaku* (“barrage”) which describes the embedded comments that originally appeared on the Japanese video-sharing site *Nico Nico* [1]. Danmu is a new genre of chat [2], and a place for online social interaction in online videos, in which viewers of the video can see previous viewers’ anonymous and undated comments towards specific points in the video. Consequently, Danmu creates a virtual atmosphere of watching together with many individuals even though the individual is looking at the screen alone [3]. Limited research on the topic has found that lonely individuals tend to stare at Danmu for longer time periods (i.e., more fixation duration and counts) when watching story-based advertisement videos [3]. Given that social interaction problems and social gratification can predict problematic online video watching behavior [4, 5], research is warranted on whether preference for Danmu (e.g., staring for longer periods at Danmu), as a potential way of seeking for social interaction and alleviating loneliness, is associated with problematic online video watching.

## Literature review

### Theories of problematic internet use

Problematic and addictive online behaviors have been widely investigated for over two decades since technological addictions and internet addiction were first proposed [6–8]. In the pathological internet use (PIU) model distinguishing between generalized and specific internet use, Davis argued that specific PIU refers to problematic behaviors on specific online applications, such as online gambling or online gaming [9]. In a more recent theoretical framework for specific internet use disorder (IUD), the Interaction of Person-Affect-Cognition Execution (I-PACE) model, Brand et al. proposed the mechanism and development of specific IUDs (e.g., addictions to online shopping, online pornography watching, online gaming or social networking) [10]. An individual’s core characteristics were considered as among the predictive variables for the development of addictive behaviors [10]. For example, higher novelty-seeking may predict addictive gambling [11], while extraversion has been positively associated with *Facebook* addiction, exercise addiction, and mobile phone addiction [12]. Moreover, addictive behaviors can be associated with an individual’s reactions (cue-reactivity) to specific external triggers or stimuli [10]. For example, individuals with high pathological online shopping tendency were found to have increased craving for buying when presented with specific cues (pictures of online shopping sites) [13]. In sum, the I-PACE model indicates that addictive behaviors could be predicted by personal characteristics and individual’s reactions towards specific stimuli (e.g., pictures). However, it is unclear whether Danmu (floating comments) could act as a stimulus of virtual social interaction and whether reactions to such stimuli can be related to addictive online video watching.

### Attentional bias and behavioral addictions

Several experimental studies have identified attentional bias in potentially addictive activities such as problematic social media use [14], alcohol overuse [15], and problematic gambling [16]. For example, Nikolaidou et al. found that problematic social media users showed significant attentional bias towards social media-related stimuli [14]. Similarly, a study by Chen et al. found that compared to non-addicted individuals, those addicted to short online videos had a higher number of eye fixations and shorter average fixation time while watching online videos, indicating greater difficulty in maintaining attention and a higher likelihood of being easily distracted [17].

However, a few studies did not find evidence for attentional bias in activities such as sports betting [18] and *Facebook* use [19]. Lole et al. found that individuals (both bettors and non-bettors) generally gaze at wagering information more than responsible gambling messages in sports betting advertisements, while no difference was found between sports bettors (either problematic or non-problematic) and non-gamblers [18]. Hussain et al. reported no significant correlation between self-reported *Facebook* addiction and eye gaze duration on different sections (social area and update area) of the *Facebook* site [19]. Therefore, based on the research evidence, it appears that attentional bias exists for some potentially addictive activities when comparing self-reported addicts with non-addicts. However, such findings are not consistent across different activities. Therefore, it is necessary to further explore attentional bias towards specific stimuli or screen sections in potentially addictive online behaviors such as online video watching.

### Problematic online video watching, social interaction and loneliness

Recent studies have suggested an association between problematic/addictive online video watching behaviors and social interaction [4, 5, 20–22]. For example, one study found that addiction to watching short videos was predicted by social anxiety and social isolation mediated by interpersonal attachment [5]. In another study, the addictive watching of *YouTube* videos was predicted by social anxiety and virtual relationships with *YouTubers* [23]. Balakrishnan and Griffiths reported that social gratification predicted *YouTube* watching inclination, which was associated with *YouTube* addiction [4]. Therefore, social interaction (e.g., social anxiety and social gratification) appears to be a predictive factor for problematic/addictive online video watching.

According to the Interaction of Person-Affect-Cognition-Execution (I-PACE) model of online addiction, the positive reinforcement formed by the satisfaction of specific online stimuli in the development of addiction can lead to a craving for specific online cues, which is an important condition for addictive behavior [10]. Danmu comments, as noted in by Chen et al. [3], can meet the social interaction needs of individuals and increase their sense of gratification while watching online videos. This could result in positive reinforcement, consequently increasing the frequency of their online video watching and the likelihood of online video watching addiction. Therefore, it is possible that individuals with a high tendency for online video watching addiction are more likely to have a higher preference for Danmu comments, which warrants further exploration. Further exploration of whether Danmu (which is a social interaction system during video watching), is associated with problematic online video watching is warranted.

Loneliness has also been found to be associated with digital addiction [24], gaming addiction [25] and internet addiction [26, 27]. The I-PACE model also suggests that loneliness could be one of the predictors of addictive online behaviors. Given such an association, lonely and addicted online video watchers might share similar habits or behavioral patterns. As aforementioned, higher loneliness was associated with longer gaze time on Danmu [3]. Therefore, it is possible that both problematic online video watchers and lonely online video watchers pay more attention to Danmu.

### Studies on Danmu

To date, empirical studies examining Danmu have focused on three aspects: the reasons for viewing Danmu, the psychological impacts of Danmu, and the personal characteristics related to Danmu viewing. This section briefly discusses these studies.

The two main purposes of media use are information-seeking and emotion fulfillment [3, 28, 29]. Research has indicated that individuals pay attention to Danmu for three reasons: information-seeking, entertainment, and social connection [30]. Information-seeking in Danmu is especially evident in online learning situations. For example, Leng et al. found that students obtained better learning outcomes when watching online courses with Danmu [31]. For the purpose of entertainment, users may try different types of expressions to express humor on Danmu. Individuals also watch Danmu during online videos in order to obtain gratification in social interaction, which is a similar mechanism to the co-viewing of television programs [30]. For example, it was reported that more than 60% of individuals used social media while watching television in seven countries and districts (Ericsson Consumer Lab, as cited in Chen et al. [p. 1]) [30], and more than 70% of primetime active *Twitter* users tweeted about television shows while watching them [32]. It appears that some individuals seek social opportunities or social rewards while watching television or videos [30, 32], although the purpose and motivations of using social media are likely to be complex. Using social media while watching television seems to satisfy individuals’ desire for socializing. Similarly, Danmu may also provide a chance for social interaction during online video watching.

Danmu has been found to have positive emotional effects on online video watchers. He and Muroi investigated the influence of Danmu on emotions using an experimental design [33]. Participants completed the PANAS (Positive and Negative Affect Scale) before and after watching videos with or without Danmu. Results showed that watching videos with Danmu increased positive emotions and reduced negative emotions [33]. Although the video content used in the study was limited (i.e., only short-form entertainment videos were shown), the findings show the potential rewarding effect of Danmu viewing. Research has also indicated that Danmu posters use various approaches (e.g., culturally-specific amusing expressions) to create Danmu humor for the other watchers [1, 2]. Therefore, watching Danmu, for both entertainment or social interaction purposes, seems to be enjoyable for some video watchers. However, Danmu can also be associated with negative emotions, such as loneliness. Using eye-tracking data, Chen et al. showed that highly lonely video watchers gazed at the Danmu area for a longer time when watching story-based ads (compared with hard-selling ads) [3]. This suggests that lonely individuals tend to pay more attention to Danmu for specific types of videos.

A few studies have reported the relationship between personality and Danmu preference. Chen et al. found that individuals with lower extraversion and higher openness preferred to watch videos with Danmu [30]. However, it seems counter-intuitive that preference for Danmu, as a means for social interaction and information seeking [30], was associated with introversion. Wolff and Kim found that extraversion and openness were the two personality traits most broadly and significantly associated with social networking behaviors (i.e., making contact with other individuals) [34].

According to the Big Five model of personality, extraversion refers to individuals who are outgoing and gregarious, while openness describes individuals who are curious and adventurous to new experiences [35]. Considering these definitions and the purposes of viewing Danmu (information-seeking, entertainment, and social connection) [30], it is possible that extraverted people who are more talkative and open could likely pay more attention to Danmu (given that it is a type of non-traditional online comment). However, studies examining the associations between key personality traits in the Big Five model (e.g., agreeableness, conscientiousness, neuroticism) and Danmu preference are limited. A recent study found that extraversion, conscientiousness and agreeableness were positively associated with activeness on *Twitter* [36]. Neuroticism was found to be associated with more negative posts on social media [37]. In other words, these three personality traits were positively associated with greater online social interaction. Therefore, it may be that agreeableness, conscientiousness, and neuroticism are also positively associated with Danmu preference.

Overall, watching Danmu appears to be associated with the desire for social interaction, positive affect, loneliness, and specific personality traits, but the empirical evidence is limited. It appears important to further explore the potential correlates (e.g., loneliness and personality) with Danmu watching, using multi-method approaches (e.g., objective eye movement tracking during video watching as well as self-reported data). Such approaches would further the understanding of online video watching behaviors, particularly in relation to potentially problematic behaviors.

### Research aims

The present study examined the relationships between Danmu preference, problematic online video watching, loneliness, and personality. Based on previous studies, it was hypothesized that:

#### H_1_

Lonely individuals would prefer to watch Danmu.

#### H_2a_

Individuals with high extraversion would prefer to watch Danmu.

#### H_2b_

Individuals with high openness would prefer to watch Danmu.

#### H_2c_

Individuals with high agreeableness would prefer to watch Danmu.

#### H_2d_

Individuals with high conscientiousness would prefer to watch Danmu.

#### H_2e_

Individuals with high neuroticism would prefer to watch Danmu.

#### H_3_

Individuals with higher levels of problematic online video watching would prefer to watch Danmu.

#### H_4_

**Fig. 1:**
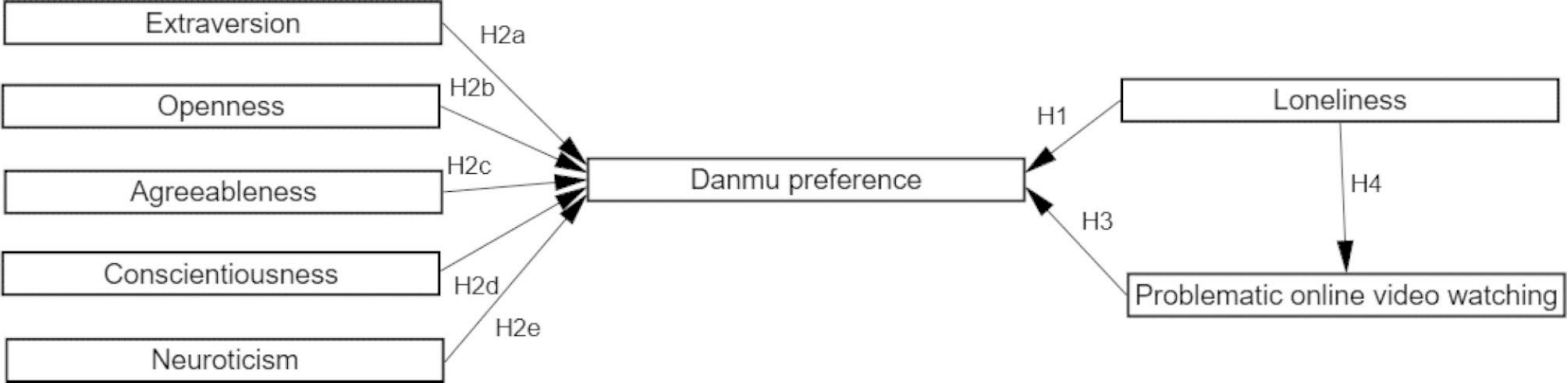
The hypothesized model

Lonely individuals would have higher levels of problematic online video watching than less lonely individuals. The hypothesized model is shown in Fig. 1:

In order to collect objective data for Danmu preference, as well as self-report data concerning preference for Danmu, the present investigation comprised two studies. More specifically, it collected both eye-tracking data of Danmu area fixation (duration and counts) and self-reported Danmu preference data. Study 1 explored the relationships between Danmu preference, problematic online video watching, personality, and loneliness based on survey self-report data. Study 2 used the same survey as that in Study 1 as well as collecting eye-tracking data including fixation duration and counts on the area of interest (i.e., the on-screen Danmu). Study 2 explored the relationships between Danmu preference, problematic online video watching, personality, loneliness, and objective Danmu area fixation (duration and counts).

## Study 1

### Participants

The sample comprised 316 participants (226 females and 90 males) from a university in Eastern China. The average age was 20.99 years (*SD* = 1.84), ranging from 18 to 29 years. The participants comprised 260 undergraduates and 56 postgraduates from different subjects including chemistry, communication, education, physics, psychology, and sociology.

### Materials

The six-item Bergen Social Media Addiction Scale (BSMAS) [38] (Cronbach alpha = 0.88) validated in simplified Chinese (Cronbach alpha = 0.73) [39] was modified to assess problematic online video watching. Items are rated from 1 (*Very Rarely*) to 5 (*Very Often*). The words “social media” were replaced with the words “online videos”. An example item is “…spent a lot of time thinking about online videos or planned use of online videos”. The Cronbach’s alpha of the modified BSMAS in the present study was 0.85.

The six-item UCLA Loneliness Scale (ULS-6) validated in simplified Chinese by [40] was used to assess loneliness (Cronbach alpha = 0.88). The ULS-6 was developed from the 20-item UCLA Loneliness Scale [41]. Items are rated from 1 (*Never*) to 4 (*Always*). An example item is “People are around me but not with me”. The Cronbach alpha of the ULS-6 in the present study was 0.90.

The 10-item short version of the Big Five Inventory (BFI-10) [42] was used to assess extraversion, agreeableness, conscientiousness, neuroticism, and openness. This study adopted the validated simplified Chinese version of the BFI-10 [43]. Items are rated from 1 (*strongly disagree*) to 5 (*strongly agree*). An example item assessing extraversion is: “I see myself as someone who is outgoing, sociable”. The Cronbach alphas of the subscales in the present study were (as expected) low ranging from 0.26 to 0.38 because of the use of just two items per personality trait. However, the developers reported that the short-form BFI has strong correlations with the BFI-44, from *r* = .74 (agreeableness) to *r* = .89 (extraversion), and sufficient test-retest reliability (r = .75).

In order to assess the preference for watching Danmu comments, participants were asked to rate their levels of agreement on the single item “I like to watch Danmu while watching online videos” from 1 (*strongly disagree*) to 5 (*strongly agree*), with higher values indicating higher preference for Danmu.

Data concerning the participants’ sociodemographic information were also collected in the survey. The questions included age, gender, year of study, and study major.

### Procedure

The survey was distributed both online and offline to the target students. The online questionnaire was hosted an online survey platform (www.wjx.cn) with a QR code created for distribution. Advertisements for the study, including the QR code and consent information, were posted on campus and on social media groups. The survey was also distributed in class using paper-based questionnaires (printed in A4 size papers) with the help of the teachers. All participants read the consent information at the beginning of the survey and volunteered to participate in this study before completing the survey. In total, 150 paper surveys were distributed and 147 responses returned with one invalid survey. The online survey resulted in 170 valid responses. Consequently, a total of 316 participants were included in the analysis. The study was approved by the ethics committee of the first author’s institution and was conducted in accordance with the Declaration of Helsinki. All participants gave their consent before starting the survey and all data were anonymized.

### Data analysis

Descriptive statistics were calculated including ranges, mean scores, standard deviations, skewness, and kurtosis. Pearson’s product-moment correlation analysis was conducted to calculate the associations between variables. Regression imputation was used to replace the missing values for structural equation modelling. Data analyses were performed using IBM SPSS version 26 and AMOS version 26. The threshold for statistical significance was *p* < .05.

### Results

Descriptive statistics for the scales are shown in Table 1. In total, 317 responses (147 paper-based and 170 online) were collected with one participant providing invalid responses. Therefore, the survey data analysis comprised 316 participants. The observed ranges show that all the scores were sufficiently distributed. Skewness and kurtosis show that all the scales were normally distributed. Participants scored 15.99 on average (*SD* = 4.60) for problematic online video watching (out of 30) and 13.11 (*SD* = 3.97) for loneliness (out of 24).

**Table 1:** Descriptive statistics for participants in Study 1 (N = 316)

Variables	No. of items	Range		Mean	SD	Skewness	Kurtosis
		Potential	Observed				
Problematic online video watching	6	6–30	6–30	15.99	4.60	0.14	0.14
Loneliness	6	6–24	6–24	13.11	3.97	0.30	0.05
Extraversion	2	2–10	2–10	5.97	1.69	0.11	− 0.18
Agreeableness	2	2–10	3–10	7.29	1.58	− 0.32	− 0.50
Conscientiousness	2	2–10	2–10	5.60	1.60	0.06	− 0.04
Neuroticism	2	2–10	2–10	6.32	1.64	− 0.08	− 0.11
Openness	2	2–10	2–10	7.27	1.68	− 0.44	− 0.31
Danmu preference	1	1–5	1–5	3.47	1.17	− 0.49	− 0.63

Pearson’s correlation analysis (see Table 2) shows that a higher level of problematic online video watching was significantly positively correlated with higher loneliness (*r* = .29, *p* < .01), neuroticism (*r* = .12, *p* < .05), self-reported Danmu preference (*r* = .19, *p* < .01), and lower conscientiousness (*r* = − .14, *p* < .05). Loneliness was significantly negatively correlated with extraversion (*r* = − .20, *p* < .01), agreeableness (*r* = − .21, *p* < .01) and conscientiousness (*r* = − .14, *p* < .05), and significantly positively correlated with neuroticism (*r* = .43, *p* < .01). Self-reported Danmu preference was significantly positively correlated with extraversion (*r* = .15, p < .01) and openness (*r* = .14, *p* < .01).

**Table 2:** Pearson’s product-moment correlations (Study 1; N = 316)

Variables	1	2	3	4	5	6	7	8
1. Problematic online video watching	-							
2. Loneliness	**0.29** ^**^	-						
3. Extraversion	− 0.01	**− 0.20** ^**^	-					
4. Agreeableness	− 0.09	**− 0.21** ^**^	**0.22** ^**^	-				
5. Conscientiousness	**− 0.14** ^*^	**− 0.14** ^*^	0.11	0.10	-			
6. Neuroticism	**0.12** ^*^	**0.43** ^**^	**− 0.20** ^**^	**− 0.16** ^**^	**− 0.20** ^**^	-		
7. Openness	− 0.07	− 0.07	**0.20** ^**^	**0.16** ^**^	**0.14** ^*^	− 0.05	-	
8. Danmu preference	**0.19** ^**^	0.05	**0.15** ^**^	0.05	0.00	0.05	**0.14** ^*^	-

*Note*. *p < .05, **p < .01 (2-tailed).

**Fig. 2:**
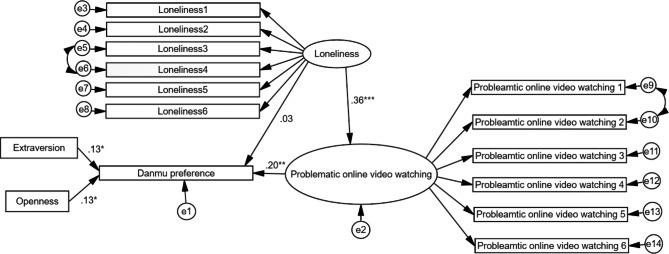
Structural equation model with path coefficients. (*N* = 316) *Note*: *p < .05, **p < .01, ***p < .001. The residuals were presented as “e” (e.g., e1, e2) in Fig. 2.

Figure 2 shows the structural equation model containing the hypotheses with path coefficients. The model fit was good and fitted the data well with acceptable indices (χ^2^/df = 2.099, CFI: comparative fit index = 0.949, TLI: Tucker-Lewis index = 0.937; RMSEA: root mean square error of approximation = 0.059; SRMR: standardized root mean square residual = 0.0652). Danmu preference was positively predicted by problematic online video watching (*β* = 0.20, *p* < .01), which supported H_3_. Loneliness positively predicted problematic online video watching (*β* = 0.36, *p* < .001), which supported H_4_. Both extraversion and openness positively predicted Danmu preference (*β* = 0.13, *p* < .05; *β* = 0.13, *p* < .05), which supported H_2a_ and H_2b_. The path from Danmu preference to loneliness was not significant in the model, meaning that H_1_ was not supported.

## Study 2

### Participants

The sample comprised 87 participants (60 females and 27 males) from a university in Eastern China (same university as Study 1). The average age was 21.17 years (*SD* = 1.96), ranging from 18 to 25 years. There were 54 undergraduates and 33 postgraduates. All participants had normal vision or normal corrected vision (with glasses) as this was a criterion for taking part on the recruitment advertisement. Study 1 and Study 2 were conducted parallel in April 2021. Therefore, the two samples comprised different participants.

### Materials

**Fig. 3:**
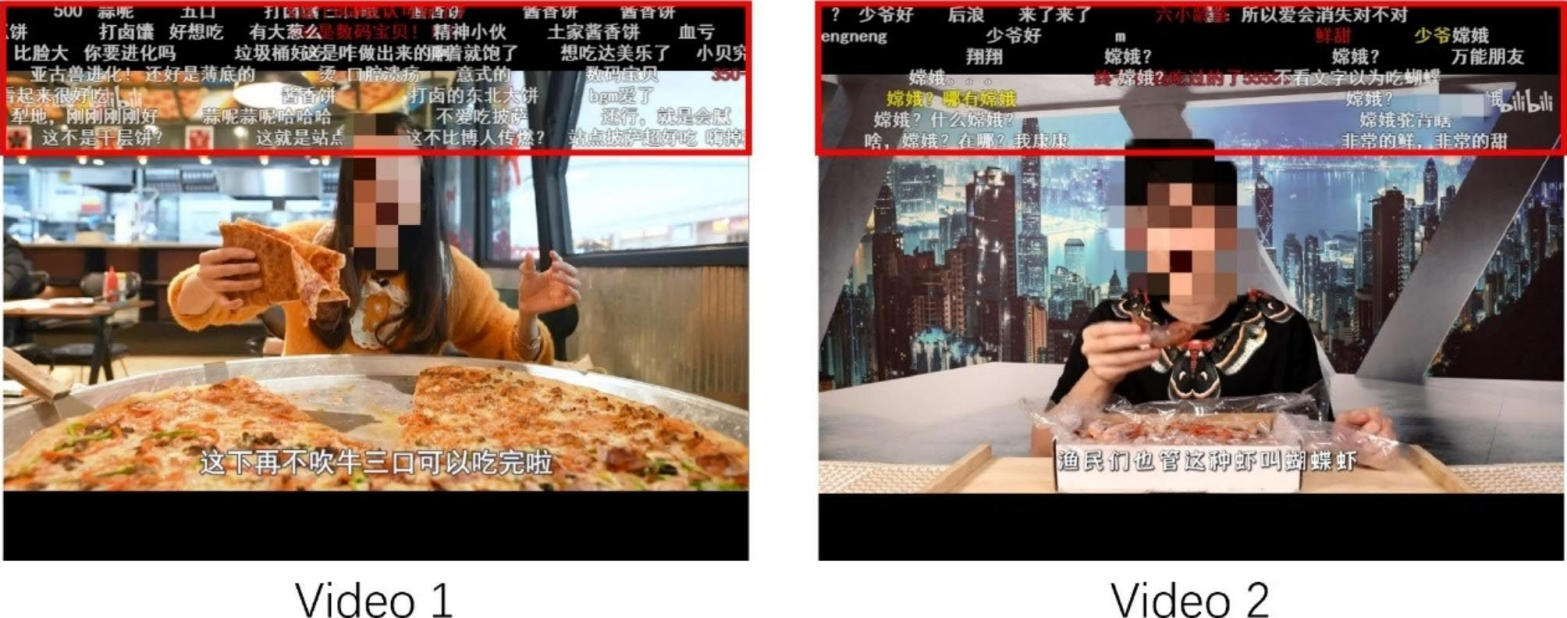
Screenshots of the selected videos and the areas of interest (AOIs) (highlighted in red boxes)

As with Study 1, Study 2 used the modified BSMAS, ULS-6, BFI-10 and the Danmu preference question to assess problematic online video watching, loneliness, personality and Danmu preference respectively. For the eye-tracking experiment, two online videos from *Bilibili.com* were selected as the material stimuli. The Danmu area, which was the area of interest (AOI) of the eye-tracking experiment, was set to be shown in the upper 1/4 of the screen (using one function on *Bilibili.com*). As shown in Fig. 3, the content of the two videos were both mukbang, where one person sits still and eats food at a table. This content was selected because the main video content (the food and the person) did not overlap with the Danmu area at most times. Video 1 comprised a female eating a big pizza. Video 2 comprised a male tasting a small plate of shrimps. For each video, only the eating parts were selected as the stimulus material (Video 1: 4 min 13 s; Video 2: 5 min 18 s). The selected video clips together with the Danmu comments were screen recorded and then imported into the Experiment Builder (EB) of Eyelink 1000 (program for eye-tracking experiment).

### Procedure

The participants were recruited via advertisements posted on campus and on social media groups. The volunteered participants were invited to come to a laboratory on campus for the eye-tracking experiment. With their consent, participants were informed with the procedure of the experiment and sat still in a chair in front of the 19-inch computer screen (*Samsung SyncMaster988MB plus*) comfortably. The screen resolution was 1280 × 1024 px and the monitor refresh rate was 85 Hz. Single eye sampling rate was 1000 Hz. The distance between the eyes and the screen was 60 cm. First, a 9-point calibration was conducted to ensure the tracker could capture the eye movements accurately. Participants were asked to fixate on a moving dot on the screen. After the calibration process (average error < 1°), the two videos were played in a randomized order. The *Eyelink* tracker recorded their eye movement data while participants watched the two videos. Finally, participants were asked to complete the survey questions and received 10 renminbi (RMB) as a reward. The whole experiment lasted approximately 20 min for each participant. The study was approved by the ethics committee of the first author’s institution and conducted in accordance with the Declaration of Helsinki. All participants gave their consent before the experiment and all data were anonymized.

### Data analysis

Eye-tracking data including AOI (Danmu area) fixation duration, AOI fixation duration percentage, AOI fixation count, and AOI fixation count percentage were obtained from *Data Viewer* software installed with *Eyelink 1000.* Descriptive statistics and Pearson’s correlation coefficients were calculated using IBM SPSS version 26. The threshold for statistical significance was *p* < .05.

### Results

#### Descriptive statistics

Descriptive statistics for the variables in the survey and the fixation data are shown in Table 3. The participants (randomly assigned with participation numbers) took part in the eye-tracking experiment and then completed the survey. The eye-tracking data and survey data were linked using the participation numbers. All data were confidential. The observed ranges for the questionnaire variables were as expected and sufficient. The skewness and kurtosis values show that all data were normally distributed. For Video 1, participants spent 30.71 s on average (*SD* = 19.32) looking at Danmu, accounting for 15.27% of their total fixation duration. Fixation counts on Danmu area for Video 1 was 131.59 times on average (*SD* = 82.31), accounting for 17.54% of their total fixation counts. For Video 2, participants spent 42.28 s looking at Danmu on average (*SD* = 29.01), accounting for 16.37% of their total fixation duration. Fixation counts on Danmu area for Video 2 was 174.96 times on average (*SD* = 111.10), accounting for 19.47% of their total fixation counts. For the two videos together, participants spent 72.23 s looking at Danmu on average (*SD* = 43.85), 15.65% of their total fixation duration. Fixation counts on Danmu area was 304.18 times on average (*SD* = 182.19), 18.42% of their total fixation counts.

**Fig. 4:**
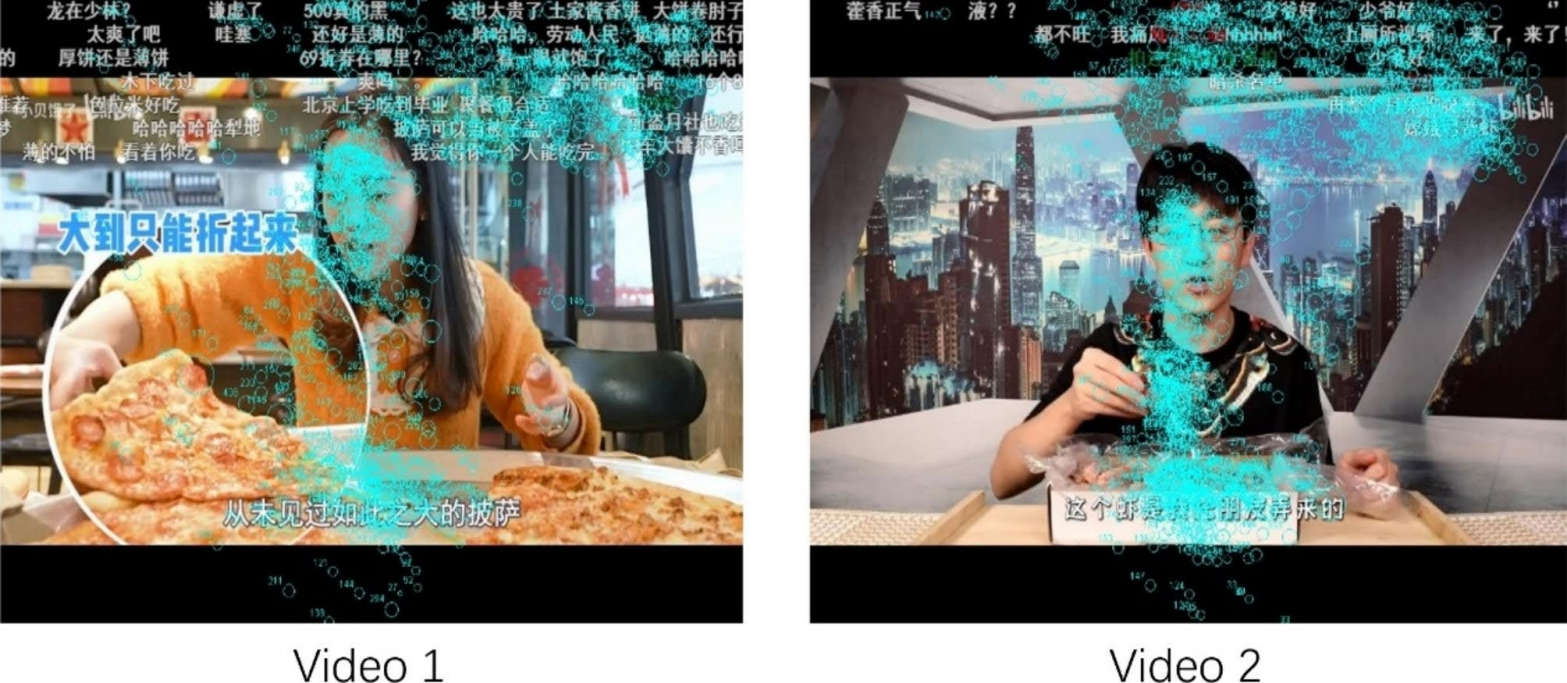
An example image of fixation data from Participant 25

Figure 4 shows one participant’s (P25) fixations on the screen for the two videos as an example. This participant spent 37.91% of her fixation time on Danmu for Video 1 and 40.32% for Video 2. Almost half of her fixations (counts) were on Danmu (45.94% for Video 1 and 46.66% for Video 2). Her scores for problematic online video watching and loneliness were high at 18 and 16 respectively.

**Table 3:** Descriptive statistics for participants in Study 2 (N = 87)

Variables	Observed range	*Mean*	*SD*	Skewness	Kurtosis
Problematic online video watching	6–23	15.78	3.69	− 0.37	0.51
Loneliness	6–20	11.99	3.50	0.07	− 0.66
Extraversion	2–10	6.30	1.64	0.31	− 0.23
Agreeableness	3–10	7.39	1.61	− 0.53	− 0.06
Conscientiousness	3–10	6.28	1.65	0.02	− 0.42
Neuroticism	2–10	6.21	1.58	− 0.09	0.08
Openness	4–10	7.94	1.49	− 0.59	− 0.42
Danmu preference	1–5	3.52	1.23	− 0.70	− 0.51
**Video 1**					
Danmu fixation duration (seconds)	1.01–79.89	30.71	19.32	0.51	− 0.44
Percentage of Danmu fixation duration	0.47-43.47%	15.27%	10.01%	0.71	− 0.07
Danmu fixation count	5-345	131.59	82.31	0.59	− 0.20
Percentage of Danmu fixation count	0.99-45.94%	17.54%	10.48%	0.45	− 0.41
**Video 2**					
Danmu fixation duration (seconds)	0.49-135.37	42.28	29.01	1.11	0.97
Percentage of Danmu fixation duration	0.19-52%	16.37%	11.01%	1.03	0.75
Danmu fixation count	3-474	174.96	111.10	0.97	0.53
Percentage of Danmu fixation count	0.42-56.08%	19.47%	11.60%	0.79	0.33
**Video 1 + Video 2**					
Danmu fixation duration (seconds)	1.50-185.11	72.23	43.85	0.72	− 0.10
Percentage of Danmu fixation duration	0.32-41.45%	15.65%	9.69%	0.84	0.27
Danmu fixation count	8-799	304.18	182.19	0.89	0.56
Percentage of Danmu fixation count	0.66-46.35%	18.42%	10.44%	0.67	0.02

#### Correlations

Pearson’s correlation analysis shows that participants with high scores on problematic online video watching, loneliness, and neuroticism tended to focus more on Danmu area, which supported H_1_ and H_3_. As shown in Table 4, problematic online video watching was positively and significantly correlated with Danmu fixation duration (*r* = .27, *p* < .05), duration percentage (*r* = 27, *p* < .05), fixation count (*r* = .24, *p* < .05) and count percentage (*r* = .27, *p* < .05) for the two videos together. This association was different between Video 1 and Video 2. Problematic online video watching was positively and significantly correlated with fixation duration (*r* = .28, *p* < .05), duration percentages (*r* = .27, *p* < .05), fixation count (*r* = .25, *p* < .05) and count percentage (*r* = .27, *p* < .05) for Video 2, and only fixation count percentage (*r* = .24, *p* < .05) for Video 1.

Loneliness was positively and significantly correlated with Danmu fixation duration (*r* = .26, *p* < .05), duration percentage (*r* = 26, *p* < .05), fixation count (*r* = .25, *p* < .05) and fixation count percentage (*r* = .22, *p* < .05) for the two videos together, which supported H_4_. Loneliness was significantly correlated with the percentages of Danmu fixation duration for both Video 1 (*r* = .23, *p* < .05) and Video 2 (*r* = .26, *p* < .05). Among the five personality traits, only neuroticism was significantly correlated with Danmu fixation. Neuroticism was positively and significantly correlated with Danmu fixation duration (*r* = .29, *p* < .01), duration percentage (*r* = 29, *p* < .01), fixation count (*r* = .30, *p* < .01) and fixation count percentage (*r* = .26, *p* < .01) for the two videos together.

Furthermore, as expected, self-reported Danmu preference was significantly correlated with eye-tracking recorded Danmu preference (*p* < .01). Different from Study 1, problematic online video watching was not significantly correlated with loneliness, the five personality traits or self-reported Danmu preference. Loneliness was negatively correlated with extraversion (*r* = − .45, *p* < .01) and agreeableness (*r* = − .42, *p* < .01), and positively correlated with neuroticism (*r* = .37, *p* < .01). Self-reported Danmu preference was positively and significantly correlated with openness (*r* = 32, *p* < .01), which was same as Study 1.

**Table 4:** Pearson’s product-moment correlations (Study 2; N = 87)

	Danmu fixation												Problematic online video watching	Loneliness	Danmu preference
	Video 1				Video 2				Video 1 + Video 2						
	Duration (ms)	%	Count	%	Duration	%	Count	%	Duration	%	Count	%			
Problematic online video watching	0.20	0.21	0.19	**0.24** ^*^	**0.28** ^*^	**0.27** ^*^	**0.25** ^*^	**0.27** ^*^	**0.27** ^*^	**0.27** ^*^	**0.24** ^*^	**0.27** ^*^	-		
Loneliness	**0.25** ^*^	**0.23** ^*^	**0.24** ^*^	0.20	**0.22** ^*^	**0.26** ^*^	**0.23** ^*^	0.20	**0.26** ^*^	**0.26** ^*^	**0.25** ^*^	**0.22** ^*^	0.14	-	
Danmu preference	**0.38** ^**^	**0.37** ^**^	**0.37** ^**^	**0.40** ^**^	**0.34** ^**^	**0.30** ^**^	**0.40** ^**^	**0.40** ^**^	**0.41** ^**^	**0.38** ^**^	**0.43** ^**^	**0.46** ^**^	0.12	− 0.03	-
Extraversion	− 0.06	− 0.03	− 0.01	− 0.01	− 0.13	− 0.11	− 0.05	− 0.07	− 0.11	− 0.07	− 0.03	− 0.04	0.12	**− 0.45** ^**^	0.16
Agreeableness	− 0.09	− 0.08	− 0.04	0.00	− 0.17	− 0.20	− 0.16	− 0.15	− 0.16	− 0.16	0.12	− 0.09	0.08	**− 0.42** ^**^	0.06
Conscientiousness	0.09	0.12	0.11	0.09	0.02	0.02	0.01	0.04	0.06	0.07	0.06	0.07	− 0.15	− 0.13	0.15
Neuroticism	**0.23** ^*^	**0.23** ^*^	0.21	0.21	**0.27** ^*^	**0.29** ^**^	**0.31** ^**^	**0.24** ^*^	**0.29** ^**^	**0.29** ^**^	**0.30** ^**^	**0.26** ^*^	0.19	**0.37** ^**^	− 0.05
Openness	0.14	0.14	0.14	0.12	0.14	0.13	0.20	0.18	0.20	0.19	0.21	0.20	− 0.10	− 0.15	**0.32** ^**^

*Note*. *p < .05, **p < .01 (2-tailed). Significant correlations are in bold.

## Discussion

### Summary of the results

Study 1 and Study 2 identified significant relationships between Danmu preference, problematic online video watching, loneliness, and personality traits using both self-report data and eye-tracking data (fixation on Danmu area while watching online videos). Participants in Study 2 spent approximately 15% of their time looking at Danmu and approximately 18% of their fixation was on the Danmu area.

In relation to the study hypotheses: participants with higher levels of loneliness in Study 2 tended to focus on Danmu more (supporting H_1_). However, H_1_ was not supported with survey data from Study 1. Study 1 found that individuals with higher levels of extraversion and openness preferred to watch Danmu more (self-reported), which supported H_2a_ and H_2b_. As with Study 1, Study 2 found that openness was significantly correlated with self-reported Danmu preference but not with the eye-tracking recorded Danmu preference. Only neuroticism was significantly correlated with actual Danmu fixation, which supported H_2e_. However, conscientiousness and agreeableness were not associated with Danmu preference, and H_2c_ and H_2d_ were not supported. Study 1 found that problematic online video watchers preferred to watch Danmu. Study 2 found that problematic online video watching was significantly correlated with more Danmu fixation (both duration and count). Therefore, the findings of both studies supported H_3_. Problematic online video watching was associated with loneliness, but only in Study 1, which supported H_4_. The different findings between the two studies may be due to the difference between the different methodologies used (i.e., self-reported and objectively recorded Danmu preference). Moreover, the much smaller sample size in the eye-tracking experimental study may have also meant a greater fluctuation in the results for the survey data collected in Study 2.

### Relationship with previous findings and implications

Study 2 found that problematic online video watching was significantly correlated with more Danmu fixation time and count. This is in contrast to Hussain et al.’s study, which found that fixation time on the social interaction area in *Facebook* was not significantly correlated with the levels of *Facebook* addiction [19]. However, the difference between *Facebook* use and online video watching needs to be noted because the comments in the Danmu area were floating across the screen in online video watching compared to fixed *Facebook* comments. It is possible that the self-perceived problematic video watchers, who might have lower levels of attention control [44], are easily distracted by the moving Danmu comments. Therefore, further research is needed to investigate the relationship between problematic online video watching and Danmu preference and fixation.

Danmu preference, problematic online video watching, and loneliness appeared to be associated with each other. Similar to previous studies and theories about problematic internet use [10, 24–27], the present study identified a significant relationship between problematic online video watching and loneliness. Previous studies have also reported a relationship between problematic online video watching and social interaction issues or needs [4, 5]. Previous research appears to show that lonely individuals tend to watch online videos problematically and require more social interaction and/or social reward. In line with this, Study 2 found that high scorers on the problematic online video watching and loneliness scales tended to stare at the Danmu area with more duration and counts. This suggests that problematic online video watchers are more likely to feel lonely and have a desire for social interaction in the Danmu area. In other words, they might try to look for virtual social interaction or the sense of being accompanied by paying more attention to the floating comments in the Danmu area. This seems to reflect the compensation mechanism in later stages of addictive behaviors as described in the I-PACE model when more rewards are needed to maintain the losing gratification [10].

Based on Study 2 (the eye-tracking experiment), problematic online video watchers tended to show more attention to the Danmu area in the videos. Therefore, online video platforms could consider adding personalized messaging relating to responsible use in Danmu in videos (e.g., *“Please take regular breaks”, “You have been continuously watching videos for five hours”*), Such messages may serve as a helpful reminder to online watchers without affecting the viewing experience, and help users to develop healthier online video watching habits. Such well-intentioned and ‘friendly’ responsible watching reminders might be more acceptable for younger age groups. However, studies to evaluate the effectiveness of such interventions would be needed.

Given the limited research evidence on Danmu and problematic/addictive online video watching, it is important to further investigate whether visual attention to Danmu and the related social interaction actions (e.g., posting or responding to previous Danmu comments) are a reflection of problematic/addictive online video watching and loneliness. This would be helpful in finding the underlying reasons for problematic online video watching from Danmu activities. It is necessary to further explore the possible links between Danmu activities and negative consequences such as addictive watching and loneliness, in order to identify potentially problematic video watchers.

Regarding personality, previous studies have found that individuals with low extraversion and high openness tend to view Danmu more [30]. In the present study, Study 1 found that participants with high openness and extraversion reported higher Danmu preference. This is probably because extraverted individuals with higher openness like to seek virtual social interaction in Danmu, which can be explained by the social purpose of Danmu viewing discussed by Chen et al. [30]. However, in Study 2, using objective eye-tracking method but with a much smaller sample size, only neuroticism was found to be significantly correlated with Danmu fixation duration and count, while no significant correlation was found for openness/extraversion and Danmu fixation/duration/count.

The different findings between Studies 1 and 2 might be due to the methodologies employed (i.e., objective vs. subjective assessment of Danmu preferences). Greater attentional bias to Danmu was associated with neuroticism but not openness or extraversion. One possible explanation might be the close relationship between neuroticism and loneliness identified in Study 2 and which has been noted in many previous studies [45]. Neurotic individuals, who are more likely to feel lonely, appear to seek social connection or the “sense of watching together” through Danmu. The reason underlying the relationship between neuroticism and Danmu fixation deserves more attention in the future, but with limited research evidence and the modest sample sizes in the present study, the personality traits related to Danmu preference remain unclear. Further research is needed on whether individuals with different personalities have different preferences for Danmu.

### Limitations and future directions

There are some limitations in the present study. First, the sample sizes were modest in both Study 1 and (especially) Study 2 (although the sample size in Study 2 would be considered large for an experiment). Although 87 participants were recruited for the eye-tracking experiment, a larger sample might produce more powerful findings. Second, the self-report measures used in both Study 1 and Study 2 might be biased because participants might have given socially desirable answers in response to survey questions. Moreover, the video content in Study 2 was mukbang (eating broadcasts), which might be another limitation. Video watchers might have different eye fixation features for other video content that are more attractive to them, for example, gaming videos for gamers. However, the overlapping of video content and Danmu comments should be considered when using other types of video content.

It should also be noted female participants outnumbered male participants in both studies could be a potential limitation. Therefore, the findings may not fully represent the experiences and perspectives of male students. Future studies in the area should recruit more gender balanced samples. Moreover, the low alpha values relating to the internal consistency of the BFI-10 is a possible limitation. The fact that there were only two items per subscale is likely to be a cause of low internal consistency at subscale level. However, the scale has been shown to have sufficient psychometric power to adequately assess individual personality traits [42, 43]. Also, the self-reported single question for Danmu preference is another possible limitation. Future studies need to use more items and/or validated psychometric scales to assess Danmu preference or viewing frequencies.

For future studies, as discussed above, different video content could be selected as materials for eye-tracking experiments for Danmu fixation. It would be interesting to explore whether video watchers view Danmu differently because of the video content only or together with the other individual factors such as problematic online video watching, loneliness and personality. Secondly, in addition to Danmu fixation, future studies could investigate the relationship between Danmu posting or replying (some Danmu comments were responses to previous ones although anonymous), and problematic online video watching has been shown to be closely related to social interaction [4, 5]. Since Danmu is a means of social interaction [30], the other social behaviors (e.g., posting and replying) not concerning fixation need more investigation. Based on the results of the present study, it seems possible that problematic online watchers or lonely watchers who cared more about Danmu would post or reply more to Danmu comments. However, more empirical evidence is needed for such speculation. It might also be interesting to investigate whether Danmu can be viewed as addictive triggers or stimuli of problematic/addictive online video watching which were described in the I-PACE model [10]. To explore this, future studies might use the cue-reactivity diagram used in previous studies on specific IUDs (e.g., pathological online shopping) [13]. Furthermore, qualitative studies on Danmu preference are needed to explore why online video watchers view or post Danmu comments in order to enhance the understanding of Danmu-related behaviors.

Future studies could also focus on the impact of AI (Artificial Intelligence)-generated Danmu comments. Ma et al. proposed a system that generates live video comments by integrating visual and textual information [46]. Their study demonstrated the effectiveness of the proposed system through extensive evaluations and showed its potential for improving user engagement in real-time video streaming platforms. It would therefore be interesting to further investigate whether such AI-generated comments are more attractive to the audience and play any role in problematic or addictive use.

## Conclusion

The present study investigated the emerging Danmu commenting system (i.e., comments that scroll across the screen) in Asian online video platforms and explored the relationships between Danmu preference, problematic online video watching, loneliness, and personality. The study is one of the first empirical investigations of Danmu and problematic online video watching using eye-tracking software. Findings showed that greater Danmu fixation (duration and counts) was associated with higher levels of problematic online video watching, loneliness, and neuroticism. As a place for virtual social interaction, Danmu appears to be more attractive to individuals who are lonely and watch online videos problematically, which supports the associations between social interaction, loneliness and IUDs noted in the I-PACE model and existing empirical evidence. This study contributes to the studies in problematic online video watching by emphasizing video watchers’ attention to virtual social interaction functions, such as Danmu. Further studies on Danmu viewing can use different types of video content for eye-tracking experiments and recruit larger samples. Interactions on Danmu (e.g., posting and replying to Danmu) also warrants further research.

## Data Availability

The datasets used and analyzed during the present study are available from the corresponding author upon reasonable request.
